# Multi-Phase Defense by the Big-Headed Ant, *Pheidole obtusospinosa*, Against Raiding Army Ants

**DOI:** 10.1673/031.010.0101

**Published:** 2010-02-18

**Authors:** Ming H. Huang

**Affiliations:** Department of Entomology, University of Arizona, Tucson, AZ

**Keywords:** *Neivamyrmex*, nest defense, phragmosis, super soldier caste, worker polymorphism

## Abstract

Army ants are well known for their destructive raids of other ant colonies. Some known defensive strategies include nest evacuation, modification of nest architecture, blockade of nest entrances using rocks or debris, and direct combat outside the nest. Since army ants highly prefer *Pheidole* ants as prey in desert habitats, there may be strong selective pressure on *Pheidole* to evolve defensive strategies to better survive raids. In the case of *P. obtusospinosa* Pergande (Hymenoptera: Formicidae), the worker caste system includes super majors in addition to smaller majors and minor workers. Interestingly, *P. obtusospinosa* and the six other New World *Pheidole* species described to have polymorphic major workers are all found in the desert southwest and adjacent regions of Mexico, all co-occurring with various species of *Neivamyrmex* army ants. *Pheidole obtusospinosa* used a multi-phase defensive strategy against army ant raids that involved their largest major workers. During army ant attacks, these super majors were involved in blocking the nest entrance with their enlarged heads. This is the first description of defensive head-blocking by an ant species that lacks highly modified head morphology, such as a truncated or disc-shaped head. *P. obtusospinosa* super majors switched effectively between passive headblocking at the nest entrance and aggressive combat outside the nest. If this multi-phase strategy is found to be used by other *Pheidole* species with polymorphic majors in future studies, it is possible that selective pressure by army ant raids may have been partially responsible for the convergent evolution of this extra worker caste.

## Introduction

Army ants are notorious for their raids of ant colonies of other species (Schneirla 1971; Mirenda et al. 1980; Gotwald 1995). During raids, army ant trails are often very direct, with a strong column trail at the base and network of trails at the swarm front (Rettenmeyer 1963; Burton and Franks 1985). The front line of these trails can span a width of 5m to 20m, depending on the army ant species (Schneirla 1971; Gotwald 1995). Captured prey is immediately dismembered while prey that gets away is usually so weakened that it becomes susceptible to parasitism or secondary predation (Gotwald 1995). Some army ants are generalists while others, such as species in the genus *Neivamyrmex,* are specialists, preying primarily on ant larvae, pupae, and/or adult workers (Gotwald 1995). Thus, there is strong selective pressure on prey to evolve effective defensive strategies against army ant raids.

When under attack by army ants, some social wasps such as *Protopolybia exigua* and *Angiopolybia pallens* exhibit vibrational alarm calls that eventually lead to synchronized nest evacuation (Chadab 1979; Chadab-Crepet and Rettenmeyer 1982). Other social wasps build nests on long, thin pedicels that are laced with ant-repellent chemicals or build nests that are completely enveloped, except for a small nest entrance where guards are present (Jeanne 1970, 1975). There are also instances where social wasps construct nests in trees that are occupied by ants that aggressively protect the tree from invading ants (Chadab-Crepet and Rettenmeyer 1982). Other social insects, such as termites, employ highly specialized soldier workers with large, biting mandibles or head nozzles that spray a glue-like substance in response to ant raids (Wilson 1971). However, under more intense raids by army ants, *Macrotermes* termites construct makeshift chambers that help protect the termite king and queen from attacks (Darlington 1985).

In the desert grasslands of the southwestern United States, army ants particularly favor *Pheidole* ants because they are highly abundant in these habitats and because most species have a relatively small body size and/or lack specific defensive strategies for army ants (Mirenda et al. 1980). In contrast, army ants tend to be deterred from invading *Pogonomyrmex, Myrmecocystus,* and *Forelius* ant colonies because of the large worker body size, physical strength, and/or defensive secretions of these species (Mirenda et al. 1980). Other ants such as *Stenamma expolitum* and *S. alas* build elevated nest entrances to minimize detection by army ants; build adjacent, normally unoccupied chambers for hiding after evacuations; and close off entrances with a single round pebble (Longino 2005). Army ant raids on *Camponotus festinatus* and *Novomessor* (*Aphaenogaster*) *albisetosus* often lead to nest evacuation (LaMon and Topoff 1981; McDonald and Topoff 1986). In contrast, *Atta* leaf-cutting ants have a minor worker caste and multiple major worker size castes (Fowler 1983). One of the defensive strategies *Atta* uses against raiding army ants is to deploy separate teams of large major workers (primary combatants) and smaller workers (assistant combatants) to counter-attack army ants outside their nest (Powell and Clark 2004). The use of large major workers as the primary defenders shows the importance of having an extra caste size. Occasionally, *Atta* leaf-cutting ants will also use soil and organic debris to plug their entrances in response to army ant attacks (Powell and Clark 2004).

The genus *Pheidole* is characterized by its dimorphic worker caste system consisting of minor workers and major workers (Wilson 2003). *Pheidole* major workers, in general, have a disproportionately greater head size than minor workers (MH Huang and DE Wheeler, in prep). The worker caste system of *P. obtusospinosa* Pergande (Hymenoptera: Formicidae) is unusual in this genus because head size varies considerably within the major worker caste. Super majors of *P. obtusospinosa* have a greater absolute head size than smaller majors, but both have a similar head size to body size ratio (MH Huang and DE Wheeler, in prep). *P. obtusospinosa* and six other New World *Pheidole* species with a similar worker caste system are all found primarily in U.S. southwestern deserts and Mexico (Wilson 2003). Interestingly, army ants in the genus *Neivamyrmex* have an overlapping geographical distribution (Gotwald 1995) and are frequently found within the vicinity of various *Pheidole* species (MH Huang, personal observation). Interactions between army ants and *Pheidole* species with polymorphic major workers have not been previously documented.

Here, the head size distribution of workers of *P. obtusospinosa* is characterized to clearly define the size ranges that represent the different worker sub-castes. The difference in head morphology of different worker castes often correlates with the ability to perform various tasks. For example, smaller workers of some ant species are more efficient at feeding brood while larger workers are more effective defenders (Hölldobler and Wilson 1990). After defining the different worker castes of *P. obtusospinosa,* field observations are reported of a successful, multi-phase nest defense strategy used by these ants against the army ant, *Neivamyrmex texanus.* In this system, only workers with the largest head size are involved in head-blocking at the nest entrance while workers of all sizes participate in aggressive combat outside the nest.

## Methods and Materials

### Worker colony demographics

A total of five *P. obtusospinosa* colonies were reared in the lab from founding queens collected in Tucson, Arizona in mid-July 2004. All colonies were kept in constant darkness, humidity, and temperature (30°C). They were sampled for major workers once in either March or April 2005, well after worker size distributions had stabilized [∼8 to 9 months after colony founding (Huang and Wheeler, unpublished)]. For each colony, all major workers were isolated into a large Petri dish, and a sample of that subpopulation was taken by randomly placing a smaller Petri dish upside-down into the larger Petri dish. All majors lying within the small Petri dish were collected and measured. The number of majors collected for each colony ranged from 76 to 111 individuals. Minor workers from each colony were sampled on different dates from major workers; three colonies were sampled for minors in February 2005 while two colonies were sampled in both February and May 2005. During each sampling date, 15 to 16 minors were randomly collected directly from each colony. A total of 446 majors and 111 minors were measured for the five colonies sampled. Head width measurements were made for both minor and major workers by using a microscope reticle. Head width was obtained by measuring the distance between the two most widely separated points on the two sides of the head, as seen from the frontal view. A cluster analysis was performed on the worker size distribution (with the assumption that there were two modes) to determine where the cutoff of the large and small major worker ranges were. The statistical package JMP 5.1 was used.

### Field observations

Observations were made between 1430 and 1530 hours Mountain Standard Time on July 2, 2006 in an oak, sycamore, and juniper forest in Gardner Canyon in Tucson, Arizona (31°42.56′N and 110°42.58′W; Elevation: 1618 meters). The observed *P. obtusospinosa* colony nested at the base of a living oak tree and had a triangular-shaped nest entrance (base: ∼ 12mm height: ∼ 9mm) partially bordered by hard tree bark and the softer soil ground surface. The head sizes of the *Pheidole* major workers involved in headblocking were estimated using measurements of army ant specimens collected in the vicinity of the head-blocking event for calibration of the photographs taken. The army ant specimens were measured using a microscope reticle. The thorax length of the army ants collected ranged from 1.6 mm to 1.85 mm (mean = 1.72 mm, S.D. = 0.096, *n* = 11). Army ant thorax length (i.e., anterior margin of pronotum down to the beginning of the first petiole) was used because this body dimension varies the least between individuals and because it was the most visible in the photographs. The estimated size of the *P. obtusospinosa* nest entrance in the photographs was also used to confirm the head sizes of *Pheidole* major workers performing head-blocking. The nest entrance size was roughly measured using the dimensions of my fingernails. The average thorax length of the army ant specimens and the estimated dimensions of the nest entrance were used together to create the scale bar (3 mm) at the bottom right corner of the photographs shown in Figure 2. This scale bar was ultimately used to estimate the head sizes of the *Pheidole* majors.

## Results

### Worker colony demographics

**Figure 1:**
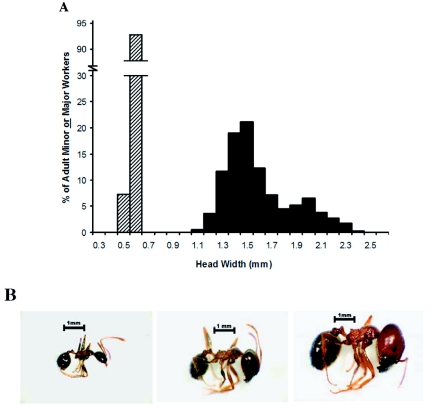
Size range and relative frequency of head width (mm) for *Pheidole obtusospinosa* [5 colonies (N_minor_ = 111; N_major_ = 446)] are shown in (a). Each lab colony was sampled once, after worker sizes stabilized. Minor workers (cross-hatched) and major workers (solid black) were sampled separately. Representative photographs of minor workers, small majors, and super majors (left to right) are shown in (b). The scale bar above each photograph is equivalent to 1 mm. High quality figures are available online. High quality figures are available online.

Minor workers of *P. obtusospinosa* have an extremely narrow size range (head width = 0.5mm to 0.7mm) and are discretely separated from major workers (Figure 1a). The major worker head width distribution was bimodal and ranged from 1.1 mm to 2.4 mm with smaller major workers present in colonies approximately three times as frequently as larger major workers. Results of the cluster analysis (assuming two modes) suggested that small majors range from 1.1 mm to 1.7 mm in head width while larger majors range from 1.7 mm to 2.4 mm. Figure lb shows that there is no major change in overall head shape when comparing small and larger majors, despite an increase in absolute head size. Here, the larger majors are referred to as super majors.

### Field observations

At the field site, a strong column of army ants (*N. texanus*) was sighted running across a dirt path toward a *P. obtusospinosa* colony at the base of a large oak tree. Initially, the army ants focused their attacks on *P. obtusospinosa* majors of all sizes outside the nest entrance. Groups of 4–6 army ants attacked the *P. obtusospinosa* majors by biting and stinging them. The *P. obtusospinosa* majors bit back with their thick, crushing mandibles. Most of the attempts by individual majors at defending themselves were futile because they were outnumbered. Meanwhile, groups of *P. obtusospinosa* minor workers tried to assist majors by stretching out the legs of individual army ants to hold them down.

**Figure 2:**
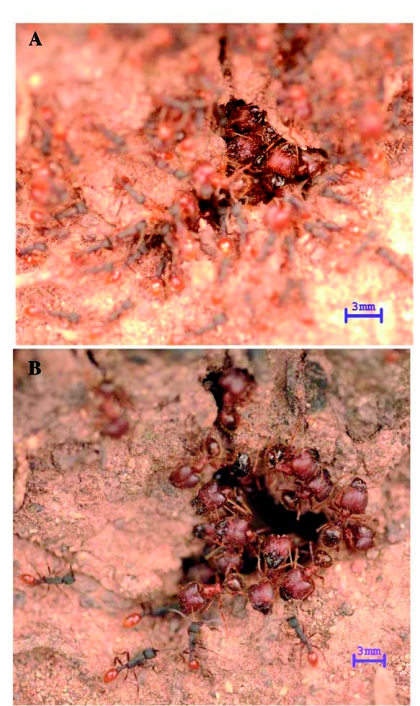
Stages of the multi-phase defensive strategy of *Pheidole obtusospinosa* against an army ant invasion: (a) headblocking behavior of *Pheidole* super majors at the nest entrance to prevent infiltration and (b) aggressive attack of *Pheidole* major workers on army ants outside the nest. The scale bar at the bottom right corner of each photo equals 3 mm. Photos by Alex Yelich. High quality figures are available online.

As the army ants attacked the *P. obtusospinosa* major workers outside the nest, the super majors guarding the entrance retreated into the nest and formed a blockade using their enlarged heads (Figure 2a); these super majors had head widths between 2 mm and 3 mm. The heads were packed tightly together with little space between them. Super majors forming the blockade remained motionless despite continuous biting and stinging attempts by army ants. Minor workers and small majors played no role in implementing the head blockade.

After failing to penetrate the nest entrance of the *P. obtusospinosa* colony, some of the army ants turned away and swarmed around the base of the oak tree, possibly trying to find another entrance into the colony. As the number of army ants at the nest entrance dwindled, the *P. obtusospinosa* super majors broke their head-blockade formation (Figure 2b) and stormed out of the nest. One group of *P. obtusospinosa* majors (large and small) attacked the army ants circling around the tree base from behind, while another group attacked the front line of the incoming army ant reinforcements by heading straight into the army ant foraging trail, occasionally dragging their abdomens on the ground. These actions of the second group of *P. obtusospinosa* majors resulted in the disorientation of army ant reinforcements at the front end of the trail.

The major sign of army ant disorientation was their change from moving in an initially straight path to moving in various, random, directions.

Without reinforcements, the group of army ants circling around the tree trunk was left behind. As this isolated group of army ants returned their attention toward the original nest entrance, both groups of *P. obtusospinosa* majors retreated into the nest, and the super majors resumed their head-blockade formation at the entrance (Figure 2a). Even after the *P. obtusospinosa* majors had abandoned the front line of the army ant trail, army ant reinforcements continued to show signs of disorientation. After 30 to 45 min of switching at least three times between the defensive head-blockade formation and the dual offensive attacks, the *P. obtusospinosa* colony drove away the raiding army ants.

## Discussion

This study shows that *P. obtusospinosa* used a multi-phase defensive strategy against invading army ants that is distinct from strategies implemented by other ants. Similar to *Atta* leaf-cutting ants, *P. obtusospinosa* has multiple major worker castes in addition to the minor worker caste (Figure 1a, b). The *Pheidole* super majors in this study played a critical role in defending the nest because they blocked nest entrances with their large heads and assisted in combating the army ants outside of the nest. In contrast, large major workers of *Atta* leaf-cutting ants have never been shown to use head-blocking (Powell and Clark 2004). Unlike *P. obtusospinosa,* the majority of *Pheidole* species are dimorphic, only having a minor worker caste and a single major worker caste with a narrow size range (Wilson 2003). Without majors with extra large heads, these species may not use blockade formation as a defensive strategy. For example, *P. desertorum* and *P. hyatti* (both have dimorphic worker caste systems) immediately evacuate their nest in response to army ant attacks (Droual and Topoff 1981; Droual 1983).

Ants such as *Colobopsis nipponicus* (Szabó-Patay 1928, as cited in Wilson 1971; Hasegawa 1993) and *Cephalotes* (= *Zacryptocerus* = *Cryptocerus*) (De Andrade and Baroni Urbani 1999) also use major workers for blocking nest entrances. Blocking of a nest entrance with the body is also known as phragmosis. In the case of *C. nipponicus* and *Cephalotes,* however, the majors have extreme modifications in head morphology for phragmosis, such as a disc-shaped or truncated head. Also, neither *C. nipponicus* nor *Cephalotes* exhibit aggressive combat outside of the nest.

Head-blocking has been suggested as a defense mechanism in other ants, such as *C.* *nipponicus* and *Cephalotes,* but this study is the first account of head-blocking in an ant species with super majors that have a noticeably enlarged head that is neither discshaped nor truncated. Therefore, extreme head modifications may not be necessary for implementing head-blocking. Having a more generalized head shape may allow *P. obtusospinosa* super majors to perform other tasks efficiently. Such additional tasks potentially include processing large food items, transporting large objects, and dismantling large enemies. The ability of *P. obtusospinosa* super majors to perform these additional tasks needs to be further investigated. In the case of *Cephalotes,* the disc-shaped heads of major workers are so morphologically specialized for head-blocking that their mandibles are reduced in size (De Andrade and Baroni Urbani 1999). As a result, they are less competent at predation, processing intact prey, and transporting large items (Wilson 1976a; Cole 1980). In addition, Powell (2008) has shown that different *Cephalotes* species have an increasingly specialized head shape as both the size of the nest entrance and the number of workers involved in head-blocking decreases. If this trend is consistent in other ant genera, *P. obtusospinosa* super majors may maintain a relatively non-specialized head shape given the relatively large size of the nest entrance and the numerous super majors involved in head-blocking, as observed in this study.

Head-blocking, however, may be a more consistently effective strategy for ants such as *Cephalotes* because they nest in dried hardwood, with the nest entrance completely surrounded by a hard substrate (Creighton and Nutting 1965; De Andrade and Baroni Urbani 1999). On the other hand, *P. obtusospinosa* has nest entrances that are surrounded by both a hard substrate such as a boulder or wood and a softer substrate such as variably loose soil. As a result, reliance on head-blocking alone in *P. obtusospinosa* may not always be effective because intruders may eventually dig through the soil. This may explain why the *P. obtusospinosa* colony observed in this study exhibited a multi-phase strategy consisting of both defensive and offensive tactics.

The behavioral specialization of *P. obtusospinosa* super majors observed here is consistent with the predictions stemming from the findings of Pie and Traniello (2007). They predict, by comparing allometric measurements of workers across various *Pheidole* species, that major workers are more behaviorally specialized than minor workers since there is a partial dissociation in head morphology between the two subcastes. Although they only examined minors and majors, observations in the present study suggest that this trend may be extended to comparisons between small major workers and super majors in species with polymorphic majors. Here, only the major workers with head widths in the largest size range (Figure 1) are involved in the specialized task of headblocking.

One aspect of the multi-phase strategy used by *P. obtusospinosa* that needs further study is how their major workers were able to cause disorientation of army ant reinforcements at the front line of the army ant trail. Since *Pheidole* majors were seen dragging their abdomens on the ground surface in the vicinity of the army ant trail, *Pheidole* majors may have altered the trail by physical or chemical manipulation. Work by Couzin and Franks (2003) has shown that the initial trailfollowing stage of the army ant *Eciton burchellii* is disordered. However, these army ant foragers are eventually able to collectively decide on a common raid direction by assessing the relative position of nest mates along the swarm trail. Assuming that *Neivamyrmex* army ants have similar microdynamic properties in trail following, manipulations of the front line of the army ant trail by *P. obtusospinosa* majors may have disrupted the interactions between individual army ant foragers, thus contributing to the overall disorientation of the army ants observed here.

Since the observations here were only based on one invasion event on one colony, behavioral experiments with more field or lab colonies of *P. obtusospinosa* are needed to determine how frequently head-blocking is implemented by *P. obtusospinosa* against army ants. In addition, the stimuli involved in coordinating the initiation and termination of head-blocking by super majors must be further examined; this defensive phase may only be implemented when the army ant raid is very intense and direct. *Pheidole dentata,* for example, can go through a sequence of up to three defensive phases against invading fire ants depending on the intensity of the invasion (Wilson 1976b). Nevertheless, this study shows strong evidence that head-blocking by super majors at the nest entrance in combination with aggressive combat outside the nest can be an effective defensive strategy, at least for the one *P. obtusospinosa* colony observed. This strategy was successful even though *P. obtusospinosa* super majors do not have extremely modified head morphology specialized for head-blocking. Although evidence is yet to be provided, it is possible that this multi-phase defensive strategy can be used effectively by other *P. obtusospinosa* colonies, as well as other *Pheidole* species with polymorphic majors, for several reasons. First, there is strong selective pressure from army ant species that have a high preference for them as prey (Mirenda et al. 1980). Second, the only seven described *Pheidole* species with super majors have a geographical distribution that completely overlaps with various army ant species (Gotwald 1995; Wilson 2003). This coexistence further increases the selective pressure on *Pheidole* ants to evolve defensive strategies against army ant raids since the likelihood and frequency of raids most likely increases with the number of predator-prey encounters. If the above is proven to be true, the observations in this study could help partially explain why *Pheidole* species with polymorphic major workers have evolved convergently in multiple occasions (Moreau 2008).
